# Thriving Community of Pathogenic Plant Viruses Found in the Human Gut

**DOI:** 10.1371/journal.pbio.0040015

**Published:** 2005-12-20

What some people won't do for science. In the early 1980s, Barry Marshall was convinced that Helicobacter pylori, not stress, caused stomach ulcers and inflammation. To convince the skeptics, he drank a straight broth of H. pylori, and promptly developed severe gastric distress. In October, he won the Nobel Prize for Physiology or Medicine (along with Robin Warren) for his efforts.

Since Marshall's experiment, it's become clear that a wide variety of bacteria, viruses, and parasites cause gastroenteritis, and that many other uncharacterized pathogens likely cause stomach flu as well. With some 100 trillion microorganisms calling the human gut home, pathogens typically represent a slim (though often raucous) minority. Bacteria, by far the most abundant resident, mostly aid digestion. Viruses in the gut come in good (bacteriophages can aid digestion and control invasive pathogens) and bad (RNA viruses) varieties. Many viral gastroenteritis pathogens were identified by analyzing patients' fecal samples, but the viruses wouldn't grow in test tubes, forcing scientists to look for other ways to generate enough viruses for study. Amazingly, they found volunteers to ingest infected stool filtrates.

Thankfully, the tools of metagenomics offer a better way. In a new study, Tao Zhang, Yijun Ruan, and their colleagues used biomolecule filtration methods and genomics to capture and characterize the global community of RNA viruses living in the human gut. The authors set out looking for pathogenic enteric viruses, but found very few human viruses. Instead, they found a “large and diverse community” of plant RNA viruses. The most abundant of these viruses infects vegetable crops, a finding that suggests that humans likely contract the virus from food—and, most surprisingly, that humans may transmit the virus to plants. [Fig pbio-0040015-g001]

**Figure pbio-0040015-g001:**
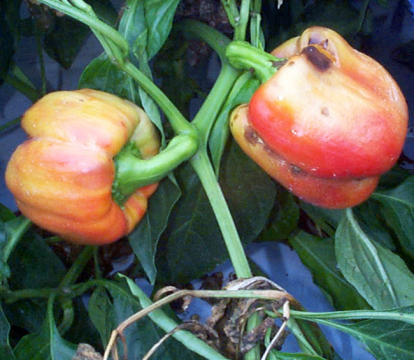
The human gut appears to harbor infectious strains of the pepper mild mottle virus (seen infecting peppers, above), suggesting that humans may serve as vector for certain plant viruses. Image: UF/IFAS Pest Alert Web site/Pamela Roberts

For their study, Zhang et al. collected three fecal samples from two healthy adults in Southern California (one volunteer supplied a second sample six months later), used filtration methods to isolate the viral particles, and then constructed three libraries (one corresponding to each sample) of nearly 37,000 sequences for analysis. Of more than 33,000 known sequences, about 75% resembled viruses, sorted into 42 different species. (The rest resembled sequences from Bacteria, Archaea, and Eukarya domains.) Only about 3% of the viral-like sequences resembled animal viruses, while 97% resembled plant viruses. Twenty-four out of 35 plant viruses identified infect commercial crops, including fruit, vegetables, and tobacco.

The most abundant plant virus, called pepper mild mottle virus (PMMV), infects a wide variety of sweet and hot peppers. When the authors compared the genetic sequences of the various PMMV strains, and then compared the sequence variation of a specific PMMV gene from all the strains, they found significant divergence at both the species and gene levels. This variability, the authors explain, suggests that PMMV exists as a diverse, dynamic population in the human gut.

So how did PMMV enter the gut? Zhang et al. enlisted three more volunteers, and tested all the food they ate two days prior to providing a fecal sample. PMMV was found in their food and at exponentially higher levels in their feces. A random test of pepper-based foods in Southern California found evidence of PMMV in three of 22 samples (none of the healthy-looking peppers tested positive). To control for local effects, the authors expanded their study to Singapore, and found PMMV in human volunteers as well as in some of the pepper-based offerings collected from food stalls. Humans not only carry this plant pathogen, we help transmit it: when Zhang et al. inoculated Hungarian wax peppers with human-borne PMMV, every one of the plants developed a PMMV infection. Since humans can transmit the virus, other animals probably can, too—suggesting that farmers with chronically infected crops might want to test any animal manure they use.

Though it's unclear how the plant viruses manage to persist inside the human gut—do they co-opt intestinal cells or use bacteria to reproduce?—the stability of PMMV suggests that it might serve as a target to help deliver vaccines or treatments aimed at intestinal disorders. What about the enteric viruses Zhang et al. originally sought? Human viruses may escape detection in healthy individuals, the authors explain, pointing to the next logical step: soliciting donations from patients with symptomatic gastroenteritis. Using the same global approach outlined here, the authors may well identify a host of other viral causes of gastric disorders. —*Liza Gross*

